# Associations between antioxidant vitamins and the risk of invasive cervical cancer in Chinese women: A case-control study

**DOI:** 10.1038/srep13607

**Published:** 2015-09-04

**Authors:** Liyuan Guo, Hong Zhu, Chengjun Lin, Jianhua Che, Xiujuan Tian, Shiyu Han, Honghui Zhao, Yumei Zhu, Dongwei Mao

**Affiliations:** 1 Department of Gynecological Oncology, Tumor Hospital of Harbin Medical University, Harbin, China; 2 Department of Obstetrics and Gynecology, The Fourth Hospital of Harbin Medical University, Harbin, China; 3 Department of Anesthesiology, The Fourth Hospital of Harbin Medical University, Harbin, China; 4 Department of Obstetrics and Gynecology, The Second Hospital of Harbin Medical University, Harbin, China; 5 Women and Children’s Hospital of Sanya, Sanya, China

## Abstract

Previous studies on the associations between dietary antioxidant vitamins and the risk of cervical cancer remain inconsistent, and little evidence is available for serum antioxidant vitamins, which provide more accurate measurements of these nutrients. We conducted a case-control study of 458 incident cases with invasive cervical cancer and 742 controls to assess the effects of diet or serum antioxidant vitamins. Higher serum antioxidant vitamins were associated with a lower risk of cervical cancer after adjusting for potential confounders. The odds ratios (ORs) for the highest (vs. lowest) quartile were 0.66 (95% confidence interval [CI] = 0.46–0.93; *P* = 0.024) for α-carotene, 0.63 (95% CI = 0.45–0.90; *P* = 0.006) for β-carotene, 0.53 (95% CI = 0.37–0.74; *P* < 0.001) for vitamin E, and 0.48 (95% CI = 0.33–0.69; *P* < 0.001) for vitamin C. Dietary intakes of vitamins E and C were inversely associated with the risk of cervical cancer. Risk of cervical cancer from serum antioxidant vitamins was more evident in passive smokers than non-passive smokers. These findings indicated that antioxidant vitamins (mainly α-carotene, β-carotene, and vitamins E and C) might be beneficial in reducing the risk of invasive cervical cancer in Chinese women, especially in passive smokers.

According to statistical data from the International Agency for Research on Cancer (IARC), in 2012 cervical cancer was the fourth most prevalent type of malignancy (62,000 new cases and 30,000 deaths) in Chinese women^1^. Although the prevalence is moderate compared with other regions, the mortality rate remains high, especially in rural areas^2^. In addition, most cases occur at 40–54 years of age, which could lead to enormous social devastation^1^. Prevention strategies are thus essential in China. Except for human papillomavirus (HPV) infection^3^, the risk factors for cervical cancer is poorly understood.

During recent decades, antioxidant vitamins have received much attention in relation to cancer prevention, particularly because they may prevent free-radical damage to DNA by neutralizing free radicals and oxidants, enhance the immune system and inhibit insulin-like growth factor (IGF)^4^,^5^.

A meta-analysis of case-control studies recently documented an inverse association between increased intake of antioxidant vitamins, such as β-carotene, vitamin E, and vitamin C, and reduced risk of cervical cancer^6^. However, the results of a prospective investigation did not support these associations^7^. These inconsistent findings might be due to the limitations of questionnaire-based assessments, including the difficulty in accurately assessing dietary intake, especially dietary vegetable and fruit intake (the primary source of dietary antioxidant vitamins) due to measurement error caused by recall bias, the non-compliance of participants, and difference in the sensitivity of individuals to questions about vegetable or fruit intake, etc. Only a few studies have used serum or plasma concentrations of antioxidant vitamins, which reflect dietary intakes, with the risk of cervical cancer or dysplasia, and the findings have been inconsistent^8^,^9^,^10^,^11^. For example, a nested case-control study in the United States showed inverse associations between cervical cancer risk and serum α-carotene and β-carotene, but not retinol nor vitamins C and E^9^. However, another nested case-control study in Finland and Sweden did not observe any associations between serum antioxidant vitamins and cervical cancer^8^. Contradictory results may also be explained by limited sample size, regional variation in antioxidant vitamins contained in foods, or other reasons such as limited sample size, confounding adjustment, etc. To the best of our knowledge, the associations between blood antioxidant vitamins and the risk of cervical cancer have not been assessed in Chinese women.

The main aim of this study was to evaluate the associations between serum antioxidant vitamin concentrations and risk of cervical cancer in a hospital-based case-control study of Chinese women. Our analyses also included effect modification by passive-smoking status because smoking is an important modification factor for antioxidant status *in vivo*^12^.

## Methods

### Study design and participants

Between July 2011 and June 2014, consecutive incident patients (diagnosed within 2 months) suffering from primary invasive cervical cancer were enrolled from three university hospitals located in Harbin, the capital city of Heilongjiang Province in northern China: the Tumor Hospital of Harbin Medical University, the Fourth Hospital of Harbin Medical University and the Second Hospital of Harbin Medical University. All cases were histologically confirmed. Two skilled pathologists independently reviewed the histology slides and confirmed diagnosis. Eligible cases were 18–70 years old at diagnosis; had received HPV DNA testing; were residents of the Heilongjiang Province for over 10 years; were alive at first contact, and could complete an interview without help. Subjects were excluded if they reported a family history of cervical cancer, significant modifications of diet within 5 years, and other major chronic diseases (e.g., diabetes, stroke and cancers) that might potentially have modified their dietary habits. Among 676 eligible cases initially recruited, 458 patients (squamous cell carcinoma: 388; adeno/adenosquamous carcinoma: 70) completed an interview and their data were included in the final analyses.

During the same period, we selected 742 control participants without cervical cancer from the same hospitals, either by screening daily census records or as recommended by doctors or nurses. They mainly came from three departments: the Department of Orthopaedics, the Department of Ophtalmology and the Department of Infectious Diseases. The selection criteria for the controls were the same as for the case subjects. All controls were hospitalized for less than 1 week with trauma (mostly fractures due to automobile accidents), influenza, orthopedic disorders (such as low back pain and disc disorders), pneumonia, or ocular fundus disease (such as glaucoma and ocular trauma). Finally, a total of 742 controls were enrolled in this study.

We obtained written consent from each study participant before the interview, and the Ethics Committee of the Harbin Medical University approved the study protocol. The methods were carried out in accordance with the approved guidelines.

### Measurement of serum antioxidant vitamin concentrations

Venous blood samples were collected after an overnight fast, separating serum within 2 hours and storing it at −80 °C until the analyses. Serum antioxidant vitamin (retinol, α-carotene, β-carotene, vitamin E, and vitamin C) assays were carried out by reverse-phase high-performance liquid chromatography (HPLC) according to the method developed by Burri *et al.*^13^, but with some modifications. In brief, 200 μl of serum samples were mixed with 500 μl 1 ml 95% aqueous ethanol containing 5 mg/l 2,6-Di-tert.-butyl-p-cresol as the internal standard, vortexed for 1 min, and then mixed with an equal volume of hexane. The hexane layer was dried under a stream of nitrogen, and the residue was resuspended in 200 μl solvent containing acetonitrile–tetrahydrofuran–methanol–ammonium sulfate (55:35:5:5, v/v). Finally, the supernatants were collected and analyzed using a C18 analytical column (Shiseido, Japan). Peaks were detected at a wavelength of 325 nm for retinol and retinyl esters by a Waters 2998 diode-array detector (Waters, USA). The coefficients of variation for the between-run assays were 4.67% for retinol, 8.22% for α-carotene, 6.81% for β-carotene, 9.92% for vitamin E, and 1.25% for vitamin C, respectively.

### Assessment of dietary intakes and other covariates

Eligible participants were interviewed by experienced interviewers using a structured questionnaire. The questionnaire items included demographic and socioeconomic factors, lifestyle habits, (family) history of chronic diseases, medical history, physical activity, and habitual dietary consumption in the previous year. Each interviewer completed questionnaires with an equal proportion of case and control subjects. Each interview took about 45 minutes to complete.

An 86-item food-frequency questionnaire (FFQ) was used to estimate average daily intake. This FFQ was adapted from the Shanghai Women’s Health Study (SWHS) and it has been validated among women in both Shanghai^14^ and Guangdong^15^. For each food item, women were asked to report the frequency (never, per day, per week, per month, and per year) and the amounts for corresponding frequency during the previous year. Data from the Chinese Food Composition Table^16^ were used for conversion to daily energy and nutrient intakes. Dietary antioxidant vitamins included retinol equivalents (animal-derived vitamin A and plant-derived β-carotene), vitamins E and C.

Patients with any first- or second- degree relatives affected by cervical or other types of cancers were considered to have a family history of cancer. Current smoking was defined as smoking ≥1 cigarette/day during the past year; Passive smoking was defined as living with someone who smokes in a room for at least 5 min every day in the previous year. Alcohol drinking was defined as having had an alcoholic drink ≥1 time per day during the past year. Information on HPV infection was collected through medical records. Physical activity was estimated as the average amount of time spent in daily activities including those related to occupation activities (e.g., occupation activities that require standing for a short or long time) and leisure-time activities (e.g., household chores, watching TV, and walking). Weight (kg) and height (m) were measured and then body mass index (BMI; kg/m^2^) was calculated.

### Statistical analysis

Kolmogorov-Smirnov test was used to assess the normality of the distributions of the continuous variables. The differences between cases and controls were compared for potential risk factors and dietary or serum antioxidant vitamin levels using t-tests for normally distributed continuous data, the Mann-Whitney U test for non-normally distributed continuous data, and chi-square tests for categorical data. The total energy intake was adjusted for dietary antioxidant vitamins using the residual method^17^. Energy-adjusted intakes or serum concentrations of antioxidant vitamins were grouped into quartiles (Q1–Q4) based on control subjects by gender, and the gender-specific cutoffs were then applied to the cases. The lowest quartile (Q1) served as the reference group.

Unconditional logistic regression analyses were used to analyze the associations between quartiles of dietary or serum antioxidant vitamins and the risk of cervical cancer. Two models were used to calculate odds ratios (ORs) with their corresponding 95% confidence intervals (CIs). In our multivariable model, we adjusted for age, BMI, marital status, education, family history of cancer, HPV infection, passive smoking, current alcohol drinking, calcium supplement use, multivitamin use, menopause, oral contraceptive use, estrogen use, physical activity, and energy intake. We used passive smoking as a covariate here because few participants were smokers in our study. All of the covariates were introduced using the forward stepwise method, and criteria for entry and non-entry of these confounders were *P* < 0.05 and *P* > 0.10, respectively.

To determine whether the associations were modified by passive smoking (yes vs. no), we stratified this factor. The potential interactions were assessed by adding multiplicative interaction terms. Sensitivity analyses were conducted to exclude smokers from the combined analyses.

The 2-sided value of *P* < 0.05 was considered statistically significant and all of the analyses were performed using SPSS version 17.0 (SPSS Inc, USA).

## Results

A total of 1200 participants (458 cases and 742 controls) were included in the analyses. The distribution of various potential risk factors between cases and controls are shown in Table 1. Compared with controls, cases were more likely to have higher family histories of cancers, HPV infection, passive smoking, and were also more likely to have lower education level, lower multivitamin, oral contraceptive and estrogen exposure, and less physical activity. Cases and controls did not differ in age, BMI, marital status, current smoking and drinking exposure, calcium supplement using, or menopause.

Table 2 shows the distribution of dietary or serum antioxidant vitamins between cases and controls. Cases had significantly lower total energy and vitamin C intakes than controls, while serum concentrations of α-carotene, β-carotene, and vitamin E were also observed to be lower in cases than in controls.

Table 3 shows the associations between serum antioxidant vitamins and the risk of cervical cancer. Univariate analysis indicated that higher serum concentrations of α-carotene, β-carotene, vitamin E, and vitamin C were associated with lower risk of cervical cancer (all *P*-trend < 0.05), but no significant association was observed for retinol (*P*-trend = 0.102). With further adjustments for age, HPV infection, passive smoking, and other covariates, significant associations attenuated but remained (Table 3). Significant inverse relationships were observed between risk of cervical cancer and α-carotene (OR = 0.66, 95% CI = 0.46–0.93; *P*-trend = 0.024), β-carotene (OR = 0.63, 95% CI = 0.45–0.90; *P*-trend = 0.006), vitamin E (OR = 0.53, 95% CI = 0.37–0.74; *P*-trend < 0.001), and vitamin C (OR = 0.48, 95% CI = 0.33–0.69; *P*-trend < 0.001).

Table 4 shows the risk of cervical cancer for dietary antioxidant vitamin intakes. Multivariate analyses indicated that dose-dependent inverse associations were only observed for vitamins E and C, and the ORs for the highest versus lowest quartiles were 0.55 (95% CI = 0.37–0.84; *P*-trend = 0.012) and 0.63 (95% CI = 0.45–0.89; *P*-trend = 0.016). There were no significant associations observed for retinol, α-carotene, or β-carotene.

In stratified analyses for passive-smoking status (Table 5), significantly favorable effects of serum antioxidant vitamins (retinol, α-carotene, β-carotene, and vitamins E and C) on the risk of cervical cancer were only observed for passive smokers and not for non-passive smokers. The *P* values for interactions were statistically significant for retinol, β-carotene, and vitamins E and C (all *P-*interaction < 0.05), but not for α-carotene (*P*-interaction = 0.222). Significant interaction was also observed for dietary vitamin E intake and passive-smoking status (*P*-interaction < 0.001; Supplementary table 1).

Sensitivity analyses also showed that after the exclusion of smokers (21 cases and 31 controls), the association between serum antioxidant vitamins and the risk of cervical cancer was similar in the remaining 458 cases and 711 controls compared with the entire sample (data not shown).

The proportion of missing values was 8.7% (40/458) for cases and 4.2% (31/742) for controls. When the missing values were replaced with the maximal or minimal level, or at random based on the distribution of corresponding factors in the non-missing data, the associations between dietary or serum antioxidant vitamins and the risk of cervical cancer did not significantly change (data not shown).

## Discussion

In this hospital-based case-control study, we showed that higher serum concentrations of α-carotene, β-carotene, vitamin E, and vitamin C were associated with lower risk of cervical cancer in Chinese women. We observed no such association for serum retinol. There was a similar protective effect for dietary vitamin E and vitamin C intake. The inverse associations between serum antioxidant vitamin concentrations and the risk of cervical cancer were much more significant in passive than non-passive smokers.

To the best of our knowledge, only four previous studies have examined the associations between serum antioxidant vitamins and the risk of cervical cancer^8^,^9^,^10^,^11^. A population-based case-control study^11^ that included 160 cases and 378 controls showed that serum β-carotene and vitamin E, but not retinol, were inversely associated with cervical cancer risk in Korean women. A hospital-based case-control study of low-income Brazilian women with 108 cases of invasive cervical cancer showed inverse associations for vitamin E^10^. A nested case-control study of US women with 50 cases and 100 controls showed a significantly lower risk of cervical cancer for α-carotene (OR for lower vs. upper tertile: 3.1; 95% CI = 1.3–7.6) and β-carotene (OR for lower vs. upper tertile: 3.1; 95% CI = 1.2–8.1), but not for retinol, nor vitamins C and E^9^. Another nested case-control study conducted in Finland and Sweden (38 cases and 85 controls) observed no associations between retinol and vitamin E and cervical cancer risk^8^. In our study, compared with those in the lowest quartile, participants with serum concentrations of α-carotene, β-carotene, vitamin E, and vitamin C in the highest quartile were associated with a 34%, 37%, 47%, and 52% decrease in the risk of cervical cancer, respectively, which was consistent with the findings from studies with larger sample sizes^10^,^11^. Null associations for vitamins C and E in these two nested case-control studies were probably due to their small sample sizes, and they may have lacked the power to detect any significant difference in risk^8^,^9^. However, we only observed a significant association for dietary vitamin E and vitamin C intake, and the risks for dietary intakes were significantly lower than those observed for serum biomarker concentrations. Significantly attenuated associations were probably due to a large random measurement error because of inaccurate assessments of dietary intake^18^.

Several biological mechanisms might explain the important role of antioxidant vitamins in preventing the development of cervical carcinogenesis. Antioxidant vitamins, such as α-carotene, β-carotene, vitamin E, and vitamin C could act as efficient scavengers of free radicals and oxidants to prevent free-radical damage to DNA^19^. Moreover, if the free radicals and oxidants were not neutralized by antioxidant molecules, inflammatory processes could lead to extensive damage to DNA proteins^20^. It has also been hypothesized that possessing antioxidant properties may protect the immune system from oxidative damage, enhance immune responsiveness, and inhibit IGF because immune cells are particularly vulnerable to oxidative stress^4^,^5^,^21^. In addition, animal experiments have suggested that supplementing vitamins A, C, and E prevents the development of preneoplastic lesions in rats^22^,^23^. Thus, antioxidant status *in vivo* may play an important role in protecting cellular function from damage.

It is well known that smoking is a traditional risk factor for cancers, and exposure to smoking has dose-dependent increased effects in the development of cancers including cervical cancer^24^,^25^. It is generally recognized that active smoking is a risk factor for cancers, but studies have also confirmed the harmful effect of passive smoking on carcinogenesis^26^. Few women are active smokers in China (3.8%)^27^. In our study the proportion was 5.3%, whereas that of passive smokers was over 60%^27^. After excluding smokers, our results were minimally attenuated, which was probably due to the few number of smokers (9 cases and 10 controls). However, we observed that exposure to passive smoking had a strong modification on the associations: stratified analyses revealed that the inverse association was stronger among those exposed to passive smoking than those who were never exposed to passive smoking, and P interactions for both α-carotene and vitamin E were 0.002, suggesting that cigarette smoking may have a synergic effect with low serum antioxidant vitamins on developing cervical cancer. A potential mechanism for this synergic effect might be that antioxidant vitamins, such as vitamin E, are known for their ability to scavenge free radicals and reduce the oxidative damage caused by cigarette smoking by blocking nitrosamine^28^,^29^.

We did not observe a protective effect of retinol on the risk of cervical cancer in the combined analyses, although the serum concentration of retinol was higher than in other populations (60.9 vs. 48.2 μg/dL among controls)^9^. However, after stratification by passive-smoking status, significant inverse association was observed in participants exposed to passive smoking. Further analysis revealed that the serum concentration of retinol was significantly higher in non-passive smokers than in passive smokers (72.4 vs. 45.1 μg/dL among controls). Studies have reported that abundant retinol might suppress these protective functions for retinol^19^, which might explain the paradox.

The limitations of this study should be acknowledged. First, the associations derived from a case-control study might be unable to infer causality. As data on diet were collected after the diagnosis of cervical cancer, case subjects may have altered their diets, resulting in a change in serum markers including serum antioxidant vitamin concentrations. We minimized this possibility by including only incident cases (within 2 months of diagnosis) and meticulously excluding individuals with a history of chronic diseases that might change dietary habits. In addition, adults generally maintain stable and long-term dietary habits^30^,^31^.

Second, our analyses were based on single measurements of serum antioxidant vitamin concentrations. We thus assumed that serum concentrations were stable in people for a sufficiently long time to play a role in preventing carcinogenic effects. Stable dietary habits partly supported this assumption. In addition, serum antioxidant vitamin concentrations may also differ over time because of day-to-day variation and long-term changes within people, and a single assessment of serum antioxidant vitamins may be susceptible to a large random error and short-term fluctuation, and thus attenuate associations. The coefficients of variation for our between-run assays were between 1.25% (vitamin C) and 9.92% (vitamin E), which suggests a moderate variability in measurements of serum antioxidant vitamins. These measures should be reliable enough to detect moderate associations.

Third, the use of hospital-based cases and controls is a potential limitation because their dietary habits and serum biomarkers may be different from those of the general healthy population in the same catchment areas, and thereby limit the representation of the study sample. The two hospitals were both major teaching and general hospitals in our region, and the distributions of major demographic characteristics (e.g., age, gender) were comparable with those of the source population^32^, thus reducing the possibility of selection bias. In addition, we only included controls hospitalized for less than 1 week for conditions not related to long-term modifications of diet (e.g., trauma, influenza, orthopedic disorders, pneumonia, or ocular fundus disease). In addition, the use of hospital controls tended to increase the comparability of information obtained from cases and controls, and thus may have caused us to underestimate the true associations^33^.

Fourth, although we adjusted for several lifestyle factors known to potentially affect cervical cancer risk and the inverse associations remained unchanged, we could not rule out the possibility of bias from residual confounding in observational study because of unmeasured lifestyle factors. Moreover, serum antioxidant vitamin concentrations, which were significantly influenced by dietary vegetable and fruit intakes, may just be markers of a healthy lifestyle in general. The participants in our study with higher serum concentrations of antioxidant vitamins tended to have healthier lifestyles, reflected in more physical activity, and included multivitamin users and a lower proportion of passive smokers. We doubt that unsuspected confounding fully explains our findings because (1) significant associations were observed with α-carotene, β-carotene, vitamin E, and vitamin C, but not with retinol – mainly deriving from vegetables and fruits, representing a healthy lifestyle^34^; (2) we saw little change in associations when we considered some main confounding, such as age, smoking status, and HPV infection status; and (3) our relatively consistent and biologically plausible finding appears to argue against a simple artifact as the source of our results.

Finally, the use of supplements such as vitamin A or vitamin C could also affect serum concentrations. Unfortunately, information about the types of supplement and frequency of use was not available to us. However, no substantial change in risk estimates was observed by simple adjustment for supplement multivitamin use (yes vs. no) or excluding those participants from the overall analyses.

In conclusion, this study provides some evidence for a protective role of the serum concentration of antioxidant vitamins (α-carotene, β-carotene, vitamin E, and vitamin C) in the etiology of cervical cancer, especially in individuals exposed to smoking. Our data confirm the importance of antioxidant vitamins in the process of carcinogenesis. The implication in terms of prevention is to encourage intake of fresh vegetables and fruits rich in antioxidant vitamins. Replication of this study in different populations may give further credence to its findings.

## Additional Information

**How to cite this article**: Guo, L. *et al.* Associations between antioxidant vitamins and the risk of invasive cervical cancer in Chinese women: A case-control study. *Sci. Rep.* **5**, 13607; doi: 10.1038/srep13607 (2015).

## Supplementary Material

Supplementary Information

## Figures and Tables

**Table 1:** Demographics, lifestyle characteristics of included participants.

Variables	Cases	Controls	*P*
N	458	742	
Age, year	47.4 ± 12.2	46.3 ± 12.0	0.126
Body mass index, kg/m^2^	22.3 ± 4.54	22.0 ± 4.10	0.238
Marital status, N(%)			0.156
Married	345 (75.3)	538 (72.5)	
Single/divorced/widowed	113 (24.7)	204 (27.5)	
Education level, N(%)			**<0.001**
Elementary school or below	254 (55.5)	282 (38.0)	
Middle school	129 (28.2)	270(36.4)	
High school or above	68 (14.8)	175 (23.6)	
Family history of cancer			**<0.001**
Yes	80 (17.5)	72 (9.7)	
No	367 (80.1)	658 (88.7)	
HPV infection			**<0.001**
Yes	368 (80.4)	74 (10.0)	
No	90 (19.6)	668 (90.0)	
Current smoker, N(%)			0.354
Yes	21 (4.6)	31 (4.2)	
No	402 (87.8)	688 (92.7)	
Passive smoker, N(%)			**<0.001**
Yes	220 (48.0)	251 (33.8)	
No	228 (49.8)	476 (64.2)	
Current drinker, N(%)			0.111
Yes	60 (13.1)	73 (9.8)	
No	388 (84.7)	603 (81.3)	
Calcium supplement use, N(%)		0.136	
Yes	125 (27.3)	217 (29.2)	
No	300 (65.5)	445 (60.0)	
Multivitamin use, N(%)			**0.005**
Yes	40 (8.7)	101 (13.6)	
No	389 (84.9)	586 (79.0)	
Menopause			0.331
Yes	185 (40.5)	289 (39.0)	
No	273 (59.5)	453 (61.0)	
Oral contraceptive use, N(%)			**0.009**
Yes	21 (4.6)	60 (8.1)	
No	402 (87.8)	612 (82.5)	
Estrogen use, N(%)			**<0.001**
Yes	18 (3.9)	85 (11.5)	
No	432 (94.3)	643 (86.7)	
Physical activity, MET•h/d	76.3 (25.4, 124.6)	95.3 (35.1, 132.2)	**<0.001**

Continuous variables described by means ± standard deviation for normally distributed continuous data and median (25th, 75th) for non-normally distributed continuous data.

**Table 2:** Serum antioxidant vitamin concentrations and dietary intakes by outcome status in Chinese women.

	Cases (N = 458)	Control (N = 742)	*P*
Dietary intakes^a^			
Dietary energy intake, kcal/d	1803 (569, 2614)	1893 (552, 2764)	**0.003**
Dietary retinol equivalents, μg/d	439 (231, 602)	458 (228, 611)	0.124
Dietary vitamin A, μg/d	226 (110, 414)	237 (104, 422)	0.098
Dietary β-carotene, μg/d	2558 (1202, 3925)	2653 (1299, 4001)	0.386
Dietary vitamin E, mg/d	8.9 (4.8, 19.8)	10.1 (5.9, 20.5)	0.065
Dietary vitamin C, mg/d	80 (32, 199)	112 (46, 243)	**<0.001**
Serum concentrations			
Serum retinol, μg/d	60.6 (39.2, 82.9)	60.9 (34.2, 89.1)	0.584
Serum α-carotene, μg/d	4.52 (3.69, 5.88)	5.21 (4.01, 6.23)	**0.008**
Serum β-carotene, μg/d	17.2 (12.2, 25.4)	18.9 (14.9, 30.0)	**0.001**
Serum vitamin E, μg/d	0.81(0.49, 1.12)	1.01 (0.65, 1.54)	**<0.001**
Serum vitamin C, mg/d	884 (547, 1123)	1001 (653, 1545)	**<0.001**

Data described by median (25th, 75th).

^a^Energy-adjusted.

**Table 3:** Odds ratio (95% CIs) of cervical cancer for quartiles of serum antioxidant vitamin concentrations in Chinese women.

	Quartiles of serum antioxidant vitamin concentrations				*P*-trend
	Q1	Q2	Q3	Q4	
Serum retinol					
N (case/control)	134/185	116/186	103/186	105/185	
Median (case/control), μg/d^a^	25.7/25.6	46.7/46.8	75.1/75.5	92.0/92.8	
Crude ORs (95% CI)	1.00	0.86 (0.62, 1.19)	0.77 (0.55, 1.06)	0.78 (0.57, 1.09)	0.102
Adjusted ORs (95% CI)	1.00	0.87 (0.63, 1.20)	0.79 (0.57, 1.10)	0.85 (0.61, 1.19)	0.258
Serum α-carotene					
N (case/control)	130/185	131/186	117/186	80/185	
Median (case/control), mg/d^a^	3.06/3.06	4.52/4.56	5.94/5.99	6.43/6.51	
Crude ORs (95% CI)	1.00	1.00 (0.73, 1.38)	0.90 (0.65, 1.24)	0.62 (0.44, 0.87)	**0.006**
Adjusted ORs (95% CI)	1.00	1.01 (0.73, 1.38)	0.93 (0.67, 1.29)	0.66 (0.46, 0.93)	**0.024**
Serum β-carotene					
N (case/control)	155/185	120/186	92/186	87/185	
Median (case/control), mg/d^a^	9.6/9.5	16.1/16.1	24.0/24.8	33.2/33.6	
Crude ORs (95% CI)	1.00	0.77 (0.56, 1.05)	0.59 (0.43, 0.82)	0.61 (0.44, 0.85)	**0.001**
Adjusted ORs (95% CI)	1.00	0.79 (0.57, 1.08)	0.62 (0.44, 0.87)	0.63 (0.45, 0.90)	**0.006**
Serum vitamin E					
N (case/control)	167/185	107/186	100/186	83/185	
Median (case/control), μg/d^a^	0.36/0.35	0.82/0.89	1.29/1.32	1.56/1.58	
Crude ORs (95% CI)	1.00	0.64 (0.46, 0.88)	0.60 (0.43, 0.82)	0.50 (0.36, 0.70)	**<0.001**
Adjusted ORs (95% CI)	1.00	0.65 (0.47, 0.90)	0.61 (0.44, 0.84)	0.53 (0.37, 0.74)	**<0.001**
Serum vitamin C					
N (case/control)	167/185	118/186	100/186	72/185	
Median (case/control), g/d^a^	492/495	796/799	1280/1286	1575/1600	
Crude ORs (95% CI)	1.00	0.70 (0.52, 0.96)	0.60 (0.43, 0.82)	0.46 (0.32, 0.64)	**<0.001**
Adjusted ORs (95% CI)	1.00	0.72 (0.53, 0.99)	0.61 (0.44, 0.85)	0.48 (0.33, 0.69)	**<0.001**

Crude and adjusted ORs (95% CI): from unconditional logistic regression models. Covariates include age, body mass index (BMI), marital status, education, family history of cancers, HPV infection, passive smoking, current alcohol drinking, calcium supplement use, multivitamin use, menopause, oral contraceptive use, estrogen use, physical activity, and daily energy intake (log-transformed).

^a^Median percentage of total energy in cases and controls.

**Table 4:** Odds ratio (95% CIs) of cervical cancer for quartiles of dietary antioxidant vitamin intakes in Chinese women.

	Quartiles of dietary energy-adjusted antioxidant vitamin intakes				*P*-trend
	Q1	Q2	Q3	Q4	
Dietary retinol equivalents					
N (case/control)	120/185	112/186	108/186	118/185	
Median (case/control), μg/d^a^	169/168	284/280	569/569	639/642	
Crude ORs (95% CI)	1.00	0.93 (0.67, 1.29)	0.90 (0.64, 1.25)	0.98 (0.71, 1.36)	0.870
Adjusted ORs (95% CI)	1.00	0.96 (0.69, 1.35)	0.96 (0.69, 1.35)	1.10 (0.79, 1.54)	0.597
Dietary vitamin A					
N (case/control)	118/185	112/186	118/186	110/185	
Median (case/control), μg/d^a^	79/80	176/170	325/329	450/451	
Crude ORs (95% CI)	1.00	0.94 (0.68, 1.31)	1.00 (0.72, 1.38)	0.93 (0.67, 1.30)	0.767
Adjusted ORs (95% CI)	1.00	0.98 (0.70, 1.37)	1.09 (0.78, 1.53)	1.06 (0.75, 1.50)	0.606
Dietary β-carotene					
N (case/control)	128/185	134/186	104/186	92/185	
Median (case/control), μg/d^a^	895/899	1921/1943	3215/3224	4196/4208	
Crude ORs (95% CI)	1.00	1.12 (0.82, 1.54)	0.82 (0.59, 1.14)	0.76 (0.55, 1.07)	**0.040**
Adjusted ORs (95% CI)	1.00	1.12 (0.81, 1.53)	0.84 (0.61, 1.17)	0.79 (0.57, 1.11)	0.080
Dietary vitamin E					
N (case/control)	158/185	108/186	120/186	72/185	
Median (case/control), mg/d^a^	3.6/3.8	6.5/6.6	15.4/16.2	22.8/23.1	
Crude ORs (95% CI)	1.00	0.68 (0.50, 0.94)	0.76 (0.55, 1.03)	0.53 (0.36, 0.79)	**0.004**
Adjusted ORs (95% CI)	1.00	0.69 (0.50, 0.95)	0.78 (0.57, 1.08)	0.55 (0.37, 0.84)	**0.012**
Dietary vitamin C					
N (case/control)	132/185	121/187	121/185	84/185	
Median (case/control), mg/d^a^	25/29	63/68	129/136	255/263	
Crude ORs (95% CI)	1.00	0.91 (0.66, 1.25)	0.92 (0.67, 1.26)	0.64 (0.45, 0.90)	**0.018**
Adjusted ORs (95% CI)	1.00	0.91 (0.66, 1.25)	0.91 (0.66, 1.25)	0.63 (0.45, 0.89)	**0.016**

Crude and adjusted ORs (95% CI): from unconditional logistic regression models; Covariates adjusted: see Table 3.

^a^Median percentage of total energy in cases and controls.

**Table 5:** Adjusted odds ratio (95% CIs) of cervical cancer for quartiles of serum antioxidant vitamin concentrations by passive-smoking status in Chinese women.

	Quartiles of serum antioxidant vitamin concentrations				*P*-trend	*P*-interaction
	Q1	Q2	Q3	Q4		
Serum retinol						**0.002**
N (case/control)	76/63	59/62	46/63	48/63		
Passive smokers	1.00	0.88 (0.55, 1.40)	0.59 (0.36, 0.95)	0.46 (0.28, 0.76)	**0.001**	
N (case/control)	55/119	57/119	56/119	60/119		
Non-passive smokers	1.00	0.96 (0.60, 1.54)	1.04 (0.65, 1.67)	1.28 (0.81, 2.03)	0.249	
Serum α-carotene						0.222
N (case/control)	67/62	66/63	54/63	33/63		
Passive smokers	1.00	0.91 (0.57, 1.44)	0.78 (0.48, 1.26)	0.47 (0.28, 0.79)	0.005	
N (case/control)	58/119	64/119	61/119	45/119		
Non-passive smokers	1.00	1.13 (0.72, 1.78)	1.02 (0.65, 1.61)	0.76 (0.47, 1.24)	0.241	
Serum β-carotene						0.002
N (case/control)	95/63	54/63	34/62	37/63		
Passive smokers	1.00	0.54 (0.34, 0.87))	0.34 (0.21, 0.56)	0.36 (0.21, 0.61)	<0.001	
N (case/control)	60/119	66/119	52/119	50/119		
Non-passive smokers	1.00	1.07 (0.69, 1.66)	0.86 (0.54, 1.36)	0.96 (0.61, 1.51)	0.637	
Serum vitamin E						0.001
N (case/control)	105/63	46/62	44/63	25/63		
Passive smokers	1.00	0.44 (0.27, 0.70)	0.40 (0.25, 0.65)	0.27 (0.16, 0.46)	<0.001	
N (case/control)	62/119	61/119	53/119	52/119		
Non-passive smokers	1.00	0.98 (0.63, 1.53)	0.85 (0.54, 1.33)	0.81 (0.52, 1.28)	0.298	
Serum vitamin C						0.004
N (case/control)	103/62	49/63	44/63	24/63		
Passive smokers	1.00	0.47 (0.30, 0.74)	0.44 (0.27, 0.71)	0.23 (0.13, 0.41)	<0.001	
N (case/control)	64/119	65/119	51/119	48/119		
Non-passive smokers	1.00	1.01 (0.65, 1.56)	0.78 (0.50, 1.21)	0.72 (0.45, 1.14)	0.096	

Adjusted ORs (95% CI): from unconditional logistic models; Covariates adjusted: see Table 3.
