# Supplementary material for: Associations between antioxidant vitamins and the risk of invasive cervical cancer in Chinese women: A case-control study
Source: Sci Rep. 2015 Sep 4;5:13607. doi: 10.1038/srep13607 (PMC4559762; doi:10.1038/srep13607)
Supplement: Supplementary Information [file srep13607-s1.doc]

**Associations between antioxidant vitamins and the risk of invasive cervical cancer in Chinese women: A case–control study**

Liyuan Guo, Hong Zhu, Chengjun Lin, Jianhua Che, Xiujuan Tian, Shiyu Han, Honghui Zhao, Yumei Zhu, Dongwei Mao

Supplementary table 1 Adjusted odds ratio (95% CIs) of cervical cancer for quartiles of dietary antioxidant vitamin intakes by passive-smoking status in Chinese women

|  | Quartiles of dietary energy-adjusted antioxidant vitamin intakes | | | | *P*-  trend | *P*- interaction |
| --- | --- | --- | --- | --- | --- | --- |
|  | Q1 | Q2 | Q3 | Q4 |
| Dietary retinol equivalents |  |  |  |  |  | 0.123 |
| N (case/control) | 64/62 | 57/63 | 51/63 | 48/63 |  |  |
| Passive smokers | 1.00 | 0.86 (0.52, 1.40) | 0.78 (0.48, 1.25) | 0.74 (0.49, 1.14) | 0.164 |  |
| N (case/control) | 50/119 | 53/119 | 55/119 | 70/119 |  |  |
| Non-passive smokers | 1.00 | 1.11 (0.70, 1.77) | 1.15 (0.70, 1.87) | 1.45 (0.93, 2.06) | 0.092 |  |
| Dietary vitamin A |  |  |  |  |  | 0.078 |
| N (case/control) | 68/62 | 54/63 | 56/63 | 42/63 |  |  |
| Passive smokers | 1.00 | 0.79 (0.49, 1.29) | 0.83 (0.51, 1.35) | 0.59 (0.36, 0.98) | 0.056 |  |
| N (case/control) | 46/119 | 54/119 | 60/119 | 68/119 |  |  |
| Non-passive smokers | 1.00 | 1.17 (0.73, 1.88) | 1.34 (0.83, 2.17) | 1.52 (0.96, 2.33) | 0.084 |  |
| Dietary β-carotene |  |  |  |  |  | 0.262 |
| N (case/control) | 63/63 | 77/63 | 46/62 | 34/63 |  |  |
| Passive smokers | 1.00 | 1.32 (0.83, 2.10) | 0.74 (0.45, 1.21) | 0.64 (0.39, 1.05) | **0.049** |  |
| N (case/control) | 59/119 | 58/119 | 56/119 | 55/119 |  |  |
| Non-passive smokers | 1.00 | 1.03 (0.65, 1.62) | 0.89 (0.56, 1.41) | 0.86 (0.53, 1.38) | 0.415 |  |
| Dietary vitamin E |  |  |  |  |  | **<0.001** |
| N (case/control) | 102/63 | 48/62 | 50/63 | 20/63 |  |  |
| Passive smokers | 1.00 | 0.40 (0.25, 0.64) | 0.45 (0.28, 0.73) | 0.21 (0.11, 0.41) | **<0.001** |  |
| N (case/control) | 53/119 | 58/119 | 65/119 | 52/119 |  |  |
| Non-passive smokers | 1.00 | 1.03 (0.66, 1.63) | 1.13 (0.73, 1.75) | 1.07 (0.63, 1.82) | 0.657 |  |
| Dietary vitamin C |  |  |  |  |  | 0.094 |
| N (case/control) | 72/63 | 57/63 | 59/62 | 32/63 |  |  |
| Passive smokers | 1.00 | 0.76 (0.47, 1.23) | 0.79 (0.49, 1.27) | 0.46 (0.27, 0.77) | **0.007** |  |
| N (case/control) | 58/119 | 62/119 | 60/119 | 48/119 |  |  |
| Non-passive smokers | 1.00 | 1.11 (0.71, 1.73) | 1.04 (0.66, 1.63) | 0.84 (0.53, 1.35) | 0.461 |  |

Adjusted ORs (95% CI): from unconditional logistic models; Covariates adjusted: see Table 3.
